# Optimising sampling regimes and data collection to inform surveillance for trachoma control

**DOI:** 10.1371/journal.pntd.0006531

**Published:** 2018-10-11

**Authors:** Amy Pinsent, T. Dèirdre Hollingsworth

**Affiliations:** 1 Department of Public Health and Preventative Medicine, Monash University, Melbourne, Victoria, Australia; 2 Big Data Institute, Li Ka Shing Centre for Health Informatics, University of Oxford, Oxford, United Kingdom; RTI International, UNITED STATES

## Abstract

It is estimated that 190 million individuals are at risk of blindness from trachoma, and that control by mass drug administration (MDA) is reducing this risk in many populations. Programs are monitored using prevalence of follicular trachoma disease (TF) in children. However, as programs progress to low prevalence there are challenges interpreting this indirect measure of infection. PCR and sero-surveillance are being considered as complementary tools to monitor low-level transmission, but there are questions on how they can be most effectively used. We use a previously-published, mathematical model to explore the dynamic relationship between TF and PCR throughout a control program and a sero-catalytic model to evaluate the utility of two cross-sectional sero-surveys for estimating sero-conversion rates. The simulations show that whilst PCR is more sensitive than TF at detecting infection, the probability of detecting at least one positive individual declines during an MDA program more quickly for PCR than for TF (for the same sample size). Towards the end of a program there is a moderate chance of a random sample showing both low PCR prevalence and higher TF prevalence, which may contribute to the lack of correlation observed in epidemiological studies. We also show that conducting two cross-sectional sero-surveys 10 years apart can provide more precise and accurate estimation of epidemiological parameters than a single survey, supporting previous findings that whilst serology holds great promise, multiple cross-sections from the same community are needed to generate the most valuable information about transmission. These results highlight that the quantitative dynamics of infection and disease should be included alongside the many logistical and practical factors to be considered in designing a monitoring and evaluation strategy at the operational research level, in order to help subsequently inform data collection for individual country programs. Whilst our simulations provide some insight, they also highlight that some level of longitudinal, individual-level data on reinfection and disease may be needed to monitor elimination progress.

## Author summary

Trachoma is a bacterial infection, which, with repeated infections over time, can lead to blindness. The WHO is aiming to eliminate trachoma as a public health problem by 2020, however at low prevalence levels the relationship between infection and disease prevalence is non-linear, making the interpretation of data from the two diagnostic tests challenging. However, it is hard to know if this is an expected outcome or a biological inconsistency. Sero-surveillance is being considered as an additional tool to understand transmission when infection and disease prevalence data provide different information. We highlight, through mathematical modelling, that a lack of strong correlation between infection and disease prevalence data at low levels of transmission seen in epidemiological data is not unexpected and demonstrate that multiple sero-surveillance surveys should be conducted from at least 2 different age groups in order to accurately estimate epidemiological parameters that will help to monitor low-level transmission.

## Introduction

Trachoma is targeted for elimination as a public health problem by 2020 by the World Health Organization. At the global level there has been a high degree of programmatic success in terms of control [1], as the established intervention strategies have been highly effective in a large proportion of endemic districts. There do, however, remain a number of districts, primarily in Ethiopia, where disease and infection remain persistent and endemic, despite long-term intervention programmes [2, 3]. Irrespective of a district or region’s current elimination status robust surveillance systems must be able to effectively monitor overall programmatic success, confirm elimination as well as re-emergence [4], however the appropriate choice of diagnostic and sampling strategy is unlikely to be uniform when trying to address each of the three aforementioned surveillance questions.

Currently polymerase chain-reaction (PCR) testing of eye-swabs and clinical examination for inflammation are the most established diagnostic tools for monitoring trachoma surveillance within the key indicator group of 1-9 year olds [5], although the vast majority of programmatic decisions are currently made based only on TF prevalence. However, an increasing number of studies are looking to assess the value of ‘alternative indicators’ (serology and PCR for trachoma surveillance), as it has been suggested that other factors may cause TF-like symptoms making it difficult to ascertain at low TF prevalence levels whether what is being observed is truly TF. Current epidemiological data suggests that following a period of intervention within a community the relationship between PCR and TF prevalence within the community becomes non-linear [6] and the results from the two diagnostics no longer correspond well with one another. Therefore it can be challenging and unclear how to interpret and explain such data in a programmatic setting [6].

As global prevalence of trachoma continues to decline it becomes increasingly challenging to identify and confirm TF cases and the cost of training graders becomes more expensive [7], therefore sero-surveillance for trachoma is also currently being evaluated as more long-term tool to monitor low-level transmission and re-emergence (in addition to PCR) [8, 9]. For sero-surveillance to be informative for understanding re-emergence it is first important to understand how serology relates to transmission intensity, and the duration of time which individuals in the population remain sero-positive, in order for us to understand what future sero-prevalence in the community will be post-elimination.

As programs approach the elimination phase and non-linearity in diagnostic outcomes become apparent or the utility of new surveillance tools needs to be evaluated, well-designed operational research is required before country specific programme surveillance recommendations can be provided. In this study, we provide two suggestions on how future data for trachoma surveillance could be collected in order to help provide insights into the dynamics of disease as population prevalence declines to help guide monitoring and evaluation. Here we evaluate how the proportion of TF and PCR positive individuals changes over the course of an intervention period and during re-emergence to assess if, or how, this impacts our probability of detecting infection or disease within a community. We assess whether these variations can be explained by the differences in the proportion of people in each state that would test positive with each of the different diagnostic tools. With our findings we suggest the types of data that could be collected to fully elucidate and understand the differences in prevalence patterns observed in these data. We then use simulated serological data to assess the identifiability of key epidemiological parameters from single and multiple cross-sections sampling a range of different age groups. Through this we advise on the optimal range of age groups to sample from in order to estimate the sero-conversion and sero-reversion rates for the population and for the key indicator group of 1-9 year olds.

## Materials and methods

### Simulating prevalence of PCR and TF

**Fig 1:**
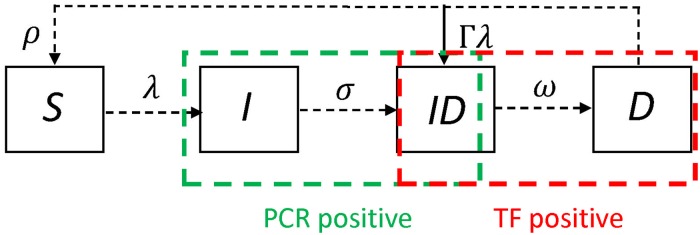
A schematic of the model structure. Individuals in the *S* state were susceptible to infection, those in the *I* state were exposed to infection and would test only PCR positive, those in the *ID* state were both PCR and TF positive, and those in the *D* state were diseased and TF only positive, but could be reinfected at a reduced rate Γ.

We simulated prevalence data within a single community of 3,000 individuals (1/3rd of which were assumed to be aged 1-9 years, denoted *N*_1_) [10, 11] to assess the probability of identifying TF and PCR positive individuals. To simulate data we used an age-structured ordinary differential equation (ODE) transmission model. We used a previously validated model structure that was identified as the most parsimonious and appropriate model when fitting to a single cross-section of age-specific PCR and TF prevalence data [12]. We used the framework of the classic *SEIR* model structure, with slightly different notation to indicate the different infection states for trachoma Fig 1. Individuals were susceptible to infection in the (*S*) state, exposed and incubating in the (*E*) state, who would test PCR positive, infected and infectious (*ID*) with detectable TF and who would also test PCR positive and those who remained diseased but were no longer infectious to others (*D*) (TF positive only), individuals in the *D* state were susceptible to re-infection with a reduced probability. Those who were re-infected then returned to the AI state (both PCR and TF positive) [12]. For each endemicity we simulated 3 annual rounds of MDA with azithromycin distributed to the whole community, assuming 80% coverage and a treatment efficacy of 85% [13].

The baseline values of the model parameters are presented in Table 1. The code for the model is available as a supplementary file.

**Table 1:** State variables, parameters definitions and values used in the model.

Name	Definition	Value	Units	Source
*S*_*i*_	Susceptible individuals	-	Number	
*I*_*i*_	Infected but not infectious individuals (PCR +ve)	-	Number	
*ID*_*i*_	Infected and infectious individuals (PCR and TF+ve)	-	Number	
*D*_*i*_	Diseased and not infectious individuals (TF+ve)	-	Number	
*β*	Transmission rate parameter	0.00655405	Proportion	
*N*_*infs*_	Maximum number of infections before immunity saturates	100	Number	[14]
*ϵ*	Degree of random mixing in the population	0.5	Proportion	[14]
*c*	Coverage	80%	Proportion	
*e*	Treatment efficacy	85%	Proportion	[13]
*λ*_*a*_	Age specific force of infection		*day*^−1^	
*σ*	Rate at which infected individuals become infectious	1/14	*day*^−1^	[15]
*ρ*_1_	Minimum rate of recovery from active disease after 1^*st*^ infection	1/300	*day*^−1^	[12, 14, 15]
*ρ*_100_	Maximum rate of recovery from active disease after 100^*th*^ infection	1/7	*day*^−1^	[12, 14, 15]
*ω*_1_	Minimum rate of recovery from infection after 1^*st*^ infection	1/200	*day*^−1^	[12, 14, 15]
*ω*_100_	Maximum rate of recovery from infection after 100^*th*^ infection	1/77	*day*^−1^	[12, 14, 15]
*α*	Infectivity of an individual proportional to their bacterial load	0.114	Proportion	[12, 14, 15]
*θ*	Rate of change of the recovery from disease rate per infection	0.3	Proportion	[14, 15]
*ϕ*	Rate of change of the recovery from infection rate per infection	0.45	Proportion	[15]
Γ	Susceptibility to re-infection in the disease state	0.5	Proportion	[12, 16]

### Testing for infection and disease

We used the transmission model to generate prevalence data at different sampling intervals to obtain the proportion of individuals PCR and TF positive at any point in time. The first scenario considered that the sampling was conducted at 6 monthly intervals over the course of 3 annual treatment rounds and we evaluated the probability (*P*_*i*_) of detecting at least 1 TF and/or PCR positive individual. The sample size used at each sampling time point was fixed across the 3 year period. The probability of identifying a PCR positive individual in a given sample collected at time *i* was the proportion of the population who we would expect to be PCR positive:

$$\begin{array}{c} P_{i}^{PCR}=\frac{E_{i}+AI_{i}}{N_{1}}\phi \end{array}$$ (1)

Where *ϕ* is the sensitivity of the assay. The probability of detecting at least one PCR positive individual was given by:

$$\begin{array}{c} 1-{\left(1-P_{i}^{PCR}\right)}^{N_{sample}} \end{array}$$ (2)

where *N*_*sample*_ was the sample size, which was used, unless otherwise stated, 50 children [11].

Only *AI* and *D* state individuals test positive for TF therefore the probability of detecting a TF positive individual was:

$$\begin{array}{c} P_{i}^{TF}=\frac{A_{i}+D_{i}}{N_{1}}\psi \end{array}$$ (3)

where *ψ* is the sensitivity of the diagnostic test for TF [17]. We note that sensitivity is a difficult parameter to quantify, particularly for TF, additionally it may reduce as local and global prevalence declines. The probability of detecting a single positive individual was similar to the expression for PCR above (Eq 2).

For the second scenario we simulated the model to endemic equilibrium for a range of TF prevalence levels (between 6% and 50%) and assessed after 3 rounds of annual MDA what the probability of detecting at least 1 PCR and TF positive individual was at the end of the intervention period only. For the final time point we also simulated sampling *N*_*sample*_ individuals from a population of individuals with this prevalence of PCR or TF, to demonstrate the range of possible outcomes which one would expect if the dynamics followed the transmission model (i.e. some correlation between PCR and TF positivity) to evaluate the range of outcomes that occur by chance.

Lastly, we assessed the probability of detecting at least 1 positive individual in a situation where infection and disease were re-emerging within the community two years post-intervention.

### Estimating epidemiological parameters from cross-sectional serology data

It has been reported that when only one sero-prevalence cross-section is available it can be challenging to estimates key parameters such as the sero-conversion rate (*λ*) and the sero-reversion rate (*ρ*) simultaneously [18]. This is because with only one cross-section is not always possible to distinguish between a scenario where people sero-convert and sero-revert quickly vs one where they sero-convert and sero-revert slowly, as both scenarios can provide comparable fits to a single cross-sectional dataset. As such, it is typically more preferable to have more than one cross-section from the sample population in order to distinguish between these two competing hypotheses.

We simulated sero-prevalence data for individuals aged 1-60 years within a community exposed to trachoma. We simulated 2 cross-sectional surveys, one pre and one post-intervention where in the post intervention data we assumed an 80% reduction in transmission occurred 10 years ago. We assumed that no individuals in the population were sero-positive as a result of exposure to any other pathogens, only trachoma. We fitted sero-catalytic models to data from both cross-sections simultaneously and also to each cross-section individually to assess how the precision and accuracy of the estimates was impacted by fitting to 1 vs 2 cross-sections. When fitting the 2 cross-sections together and the post-intervention only cross-section we estimated 4 parameters the: sero-conversion rate (*λ*), sero-reversion rate (*ρ*), the proportional drop in transmission (*γ*) and the time at which the drop in transmission occurred (*T*_*c*_).

Sero-negative individuals become sero-positive at a rate *λ* and sero-positive individuals become sero-negative at a rate *ρ* [19]. Thus the proportion of sero-positive individuals within the cross-section collected is determined by the following:

$$\begin{array}{c} \frac{dP}{dt}=\lambda \left(t\right)\left(1-P\right)\rho P \end{array}$$ (4)

Where in a model that assumes a change in transmission at an instantaneous point in time *λ* is defined as follows:

$$\begin{array}{c} \lambda \left(t\right)=\{\begin{array}{c} \lambda_{0}t<T_{c}\\ \lambda_{c}\geq T_{c} \end{array} \end{array}$$ (5)

For the pre-intervention dataset we only estimated 2 parameters *λ* and *ρ*. We also estimated epidemiological parameters from data collected from only 1-9 year olds (the key indicator group for surveillance), from both cross-sections simultaneously and individually. We then assessed how sampling an additional age group as well the current indicator group impacted the accuracy and precision of parameter estimation. We henceforth define accuracy in terms of parameter estimation as how close the paramater estimate was to the true simulated value, and precision as the narrowness of the credible intervals (CrI) for the estimate of any given parameter.

## Results

### Probability of detecting PCR and TF positives during an intervention period with a fixed sample size

**Fig 2:**
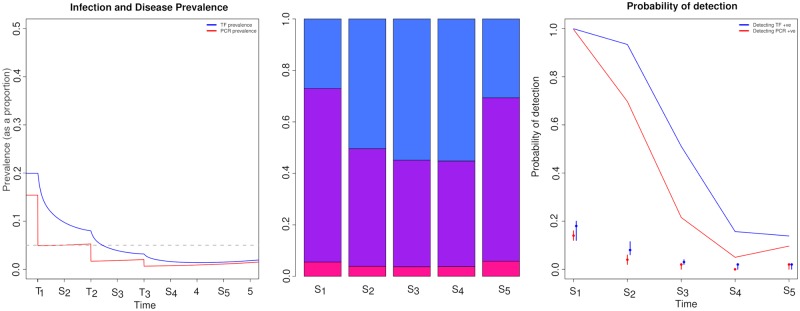
Changes in the prevalence and detectability of PCR (red) and TF (blue) positive individuals over an intervention period. a) prevalence of infection and disease prevalence change during an intervention within the community, points on the x axis labelled with an S indicate that a sample was taken at that point, and those labelled with a T indicate when treatment occurred. b) the proportion of individuals present in each diagnostic state at each sampling point during the intervention period: pink—PCR positive only, purple—PCR and TF positive, blue—TF positive only and c) indicates the probability of detecting at least 1 positive individual when taking 50 samples by PCR (red) and TF (blue) eye examination, dots represent the median prevalence point from 100 binomial samples for PCR and TF at each sampling point, the intervals represent the lower and upper inter-quartile range of prevalence.

We considered a community with a true endemic disease prevalence of 20% (16% infection prevalence). Following a single round of treatment, the prevalence of PCR detectable infection dropped much more quickly than the prevalence of TF (Fig 2a). Thus, declines in TF prevalence lagged behind the changes observed for PCR. This was consistent for all three rounds of MDA (Fig 2a). Consequently, true TF prevalence was consistently higher than true PCR prevalence within the period evaluated.

The proportion of individuals that were prevalent by any diagnostic test (Fig 2b) prior to MDA commencing showed that 6% of exposed individuals would have tested PCR-only positive, 67% would have tested PCR and TF positive, while 27% would have tested TF-only positive (Fig 2b). Once the intervention had begun the ratio of individuals in different infection and disease states altered (Fig 2b). As the intervention period progressed the proportion of individuals that tested PCR-only positive fell from 6% to 3.5%, while the proportion of individuals that tested both PCR and TF positive declined substantially from 67% to 41%. By contrast the proportion of TF-only positive people increased markedly from 27% to 55% (Fig 2b). The large decline in the overall proportion of people PCR positive helps to explain the marked reduction in the probability of detection for PCR positive individuals (Fig 2c) and the slower decline in the probability of detection of TF positive individuals (Fig 2c). As such, the opportunity to identify individuals who were both TF and PCR positive was most likely to occur when the ratio of PCR to TF positive individuals were similar in the population, which was most likely to occur at endemic equilibrium.

Once sampling time point 5 was reached we saw a slight indication that re-emergence may be occurring (Fig 2a). For sampling point *S*_5_ in comparison to sampling point *S*_4_ there was a marked increase in the proportion of individuals that tested PCR and TF positive (41% to 63%, Fig 2b) and a decrease in the proportion of individuals that tested TF-only positive (55% to 30%, Fig 2b)—these differences were reflected in the increase in probability of detection for PCR, but only a minor increase in the probability of detection of TF positives (Fig 2c).

When sampling 50 individuals at each time point, we found that with the exception of time point *S*_4_ the expected median prevalence was consistently higher for TF than PCR (Fig 2c), however the variance in the expected TF prevalence was larger than for PCR. Additionally, the lack of overlap in the prevalence estimates over the intervention period suggested that at multiple times during the intervention period it is possible that people will test positive with one diagnostic but not the other (Fig 2c). This coupled with a marked reduction in the probability of detection as prevalence declines suggests non-linearity in the results from different diagnostics is not unexpected.

### Probability of detecting PCR and TF positives during re-emergence with a fixed sample size

**Fig 3:**
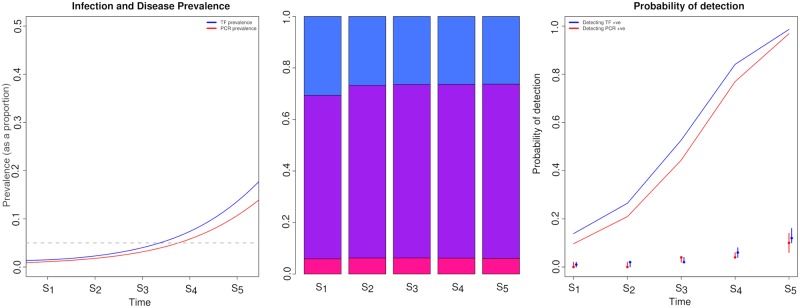
Changes in the prevalence and detectability of PCR (red) and TF (blue) positive individuals if re-emergence was occurring 18 months after the last round of MDA. a) prevalence of infection and disease prevalence change during re-emergence within the community, points on the x axis labelled with an S indicate that a sample was taken at that point, and those labelled with a T indicate when treatment occurred. b) the proportion of individuals present in each diagnostic state at each sampling point during the intervention period: pink—PCR positive only, purple—PCR and TF positive, blue—TF positive only and c) indicates the probability of detecting at least 1 positive individual when taking 50 samples by PCR (red) and TF (blue) eye examination, dots represent the median prevalence point from 100 binomial samples for PCR and TF at each sampling point, the intervals indicate the lower and upper inter-quartile range of prevalence.

Following 2 years of MDA cessation in the community we considered the dynamics of detection during a potential resurgence (Fig 3a). As re-emergence continued the rate at which TF prevalence increased was faster than that of PCR prevalence, this was because only few people test PCR-only positive, but there was an increase in the number of people who test positive PCR and TF as well as TF positive only. This was likely to be because when prevalence begins to increase gradually the rate of re-infection in the TF only state is low, due to an initial low force of infection in the community. Assessing the proportion of individuals by diagnostic state when re-emergence first began, 5.5% of exposed individuals would have only tested PCR positive, 67% would have tested PCR and TF positive, while 27% would have tested TF positive only (Fig 3b). As re-emergence continued to occur across the first 4 sampling time intervals the proportion of TF only positive individuals was consistently higher than PCR and TF positive individuals, at sampling time point 4, 41% of individuals tested PCR and TF positive, while 55% of individuals were TF positive only. In contrast, at sampling time point 5 the ratio of individuals in different diagnostic states was more comparable to that seen in the community prior to MDA being implemented (Fig 2b) where 63% of individuals tested PCR and TF positive and 30% of individuals were only TF positive (Fig 2b). As re-emergence continued the probability of detection with both diagnostics increased, the probability of detection increased at a similar rate as time progressed for both tests (Fig 3c), in contrast to the results seen when prevalence was declining during the MDA programme where the probability of detecting PCR positive individuals declined much more rapidly than TF positive individuals (Fig 2c). The variance in the estimated PCR and TF prevalences were slightly higher for PCR detectable infection, whilst the variance in the estimated prevalence of TF and PCR overlapped at some sampling time points, this was not consistent for all sampling points. Highlighting that in a low prevalence re-emergence setting it’s possible we would find individuals PCR and or TF positive (Fig 3c).

### Ratios of different diagnostic states across a range of TF prevalence levels

**Fig 4:**
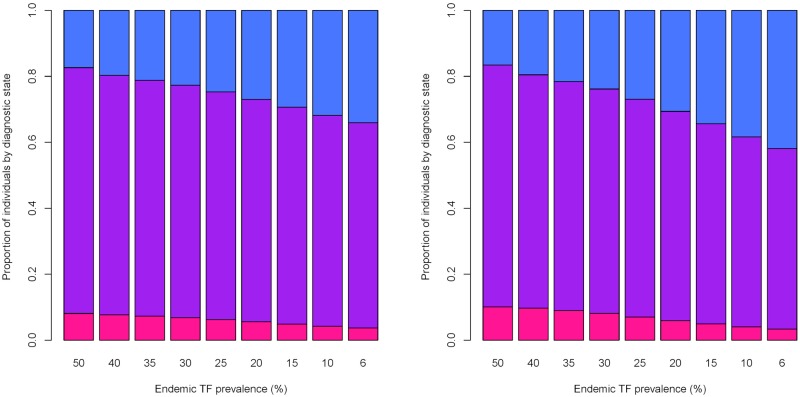
Changes in the proportion of individuals positive in each diagnostic state, pre and post MDA for different initial endemic levels of TF prevalence. On the left is the proportion of individuals positive in each diagnostic state prior to MDA occurring: pink—PCR positive only, purple—PCR and TF positive, blue—TF positive only, each x axis label indicates what the initial endemic prevalence was in the community prior to intervention. On the right is the proportion of individuals in each diagnostic state following 3 annual rounds of MDA for each initial level of endemic prevalence (as shown on the x label), again pink—PCR positive only, purple—PCR and TF positive, blue—TF positive only. At high levels of initial endemic prevalence we see the highest proportion of individuals test both PCR and TF positive as a result of rapid rates of re-infection within the community, while at lower levels of prevalence a high proportion of TF only individuals were present due to lower rates of re-infection. Changes in the proportion of individuals positive in each diagnostic state were more apparent for initial endemic prevalence’s lower than 25%.

In the pre-MDA setting at higher levels of TF prevalence the overall proportion of PCR and TF positive individuals was much higher than at lower levels of endemic prevalence. When TF prevalence was 50%, 74.5% of infected individuals were PCR and TF positive, but when TF prevalence was 10%, 64% of individuals were TF and PCR positive (Fig 4a). Here the proportion of TF-only positive individuals increased from 20%, to 32% when endemic prevalence was 50%, in comparison to 10% (Fig 4a). Whilst when TF prevalence was 30% the ratio of individuals in each diagnostic state was more comparable to when TF prevalence was 50%: (6.8%, 70%, 22%) (Fig 4a). At high levels of endemic prevalence we would expect the greatest proportion of individuals in the population to be both TF and PCR positive because individuals in the TF only state will be continuously re-infected. However, at lower levels of prevalence the rates of re-infection are not as high, resulting in a higher proportion of TF-only positive individuals.

Across all TF prevalence levels post-MDA a comparable ratio of individuals in each diagnostic state to the pre-MDA levels was observed, although for a number of initial prevalence levels the proportion of PCR only positive individuals was slightly higher than at endemic equilibrium. For example, the proportion of PCR positives only increased from 7.6% to 9.7% (Fig 4b). For lower levels of endemic prevalence post-MDA we observed a slight decrease in the proportion of individuals PCR and TF positive, and a small increase in the proportion of individuals who would test only TF positive—for an endemic TF prevalence of 10% the proportion of individuals TF and PCR positive dropped from 64% to 54.5% and the proportion of TF only positive individuals increased from 31% to 38% (Fig 4b).

Our simulations have suggested that the expected proportion of individuals detectable as both PCR and TF positive declines as the overall prevalence in the community declines, typically as prevalence declines a higher proportion of individuals become TF-only positive. Additionally, the probability of detecting an individual as PCR positive during an intervention period declines much more quickly than for TF, this difference in the probability of detection may also help to explain disparities in the reported prevalence of infection and disease as transmission declines when surveys are conducted. To understand more clearly what is happening when we observe non-linearity in prevalence in 1-9 year olds by PCR and TF surveillance we would need individual level data on PCR and TF prevalence, this would enable us to see whether the proportion of PCR and TF positives in the data is comparable to the ratios that the model predicts.

### Assessing TF prevalence vs PCR prevalence across the different levels of endemicity

**Fig 5:**
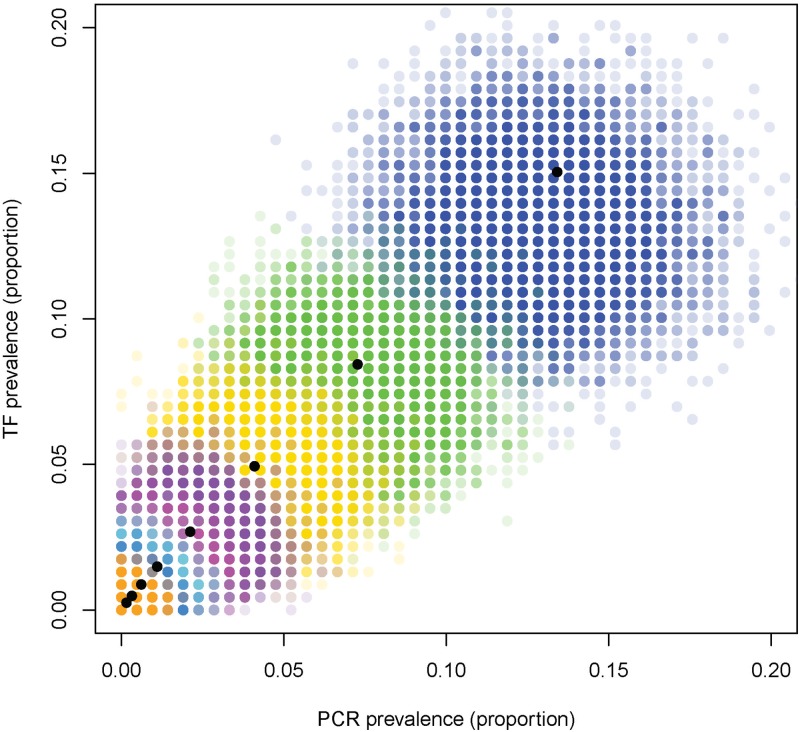
PCR and TF prevalence post-MDA for different initial endemicities (black dots), and for 10,000 different samples of 200 individuals (coloured dots) from a population with a true prevalence indicated by the black dots, for each of the different endemicity levels.

At both high and low levels of transmission the simulations above suggest that the true underlying PCR and TF prevalence levels do correlate with one another. At high levels of infection and transmission PCR and TF prevalence correlate with one another due to rapid rates of reinfection occurring, ensuring that PCR and TF prevalence correlate well with one another. As prevalence declines although the true underlying prevalence’s may correlate at low prevalence sampling noise can play an important role, leading to some samples being collected in which TF prevalence is much higher than PCR prevalence (Fig 5). For these simulations, both TF and PCR sensitivity mean that prevalence is usually underestimated (the coloured dots are down and to the left of the black dot indicating true prevalence). If sensitivity declines as prevalence continues to fall, then this discrepancy will be larger.

### Estimating epidemiological parameters from serological data

**Fig 6:**
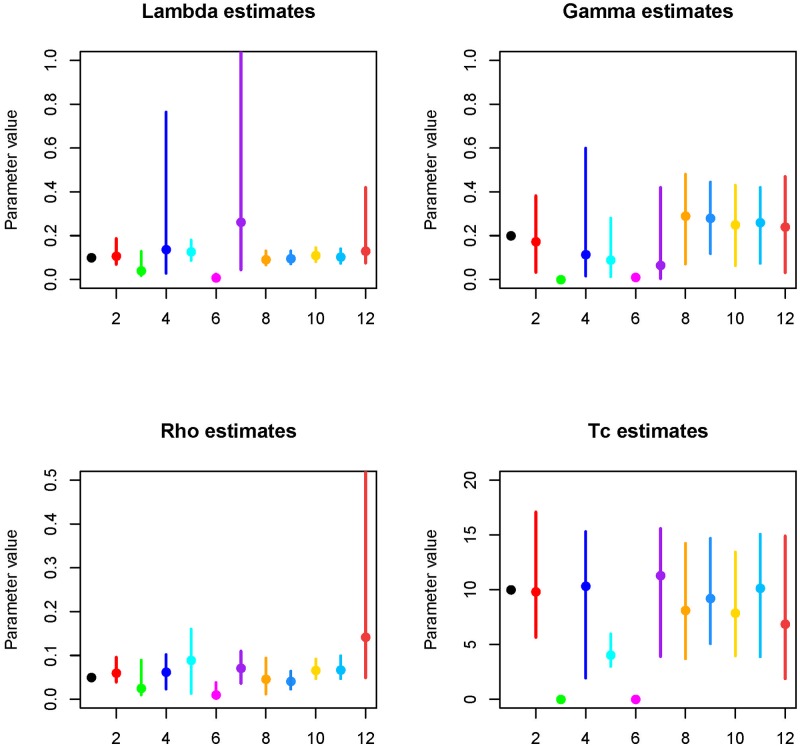
The estimated value for each sero-catalytic model parameter using different simulated datasets. For each parameter the true value is indicated in black (x label 1), fitting to all-age data for both cross-sections simultaneously is highlighted in red. All-age pre-intervention only data (green, 2 parameters estimated), all-age post intervention data only (blue), 1-9 year old data—2 cross-sections (cyan), 1-9 year old data pre-intervention data (pink), 1-9 year old data post-intervention (purple), 1-9 and 10-20 year olds pre and post-intervention (orange), 1-9 and 20-30 year olds pre and post-intervention (light blue), 1-9 and 30-40 year olds pre and post-intervention (yellow), 1-9 and 40-50 year olds pre and post-intervention (sky blue), 1-9 and 50-60 year olds pre and post-intervention (rust).

Fitting a 2 parameter model to pre-intervention cross-sectional data the median estimates of *λ* and *ρ* were lower than the true values of the simulated data: 0.04 and 0.02 vs 0.10 and 0.05, however the credible intervals included the true value (Fig 6). Fitting the post-intervention dataset in isolation the estimate of *λ* was close to the true value (0.13 vs 0.10), however the credible intervals were much wider than when the two cross-sections were fitted simultaneously. The median estimate of *γ* was lower than the true value, with much wider credible intervals in comparison to when 2 cross-sections were fitted together (Fig 6). Estimates of the *ρ* and *T*_*c*_ were similar to the true values, however the precision of the estimates were less than when 2-cross sections were fitted simultaneously (Fig 6).

Fitting 2 cross-sections to data from only 1-9 year olds (300 samples) the median estimated *λ* was similar to the true estimate (0.12 vs 0.10), *T*_*c*_ was also estimated relatively accurately. The estimate of *γ* was much lower than the true value 0.09 (CrI: 0.01-0.28), but the credible intervals did include the true value. The estimate of *ρ* was higher than the true value (0.09 vs 0.05), but the wider credible intervals still included the true value.

Fitting to pre-intervention period data from 1-9 year olds, estimates of *λ* and *ρ* were much lower than the true values and the credible intervals did not include the true values, estimates were also much lower than when a single cross section for the full dataset was fitted to, suggesting that fitting to a small cross-section of the population is not sufficient to accurately estimate these parameters. For the post-intervention data in 1-9 year olds *λ* was over-estimated and the credible interval range was large, much wider than when the full single cross section was evaluated. Estimates of *ρ* were similar when 1-9s were evaluated as when the full cross-section was, however this is likely to have traded off with the estimate of *ρ*. The median estimate of gamma was below the true value but similar to when all data was fitted to for the single post-intervention dataset, whilst *T*_*c*_ was above the true value. Therefore overall for the pre-intervention data estimates of *λ* and *ρ* were markedly different to the full dataset and when the two cross-section were fitted together. For the post-intervention data estimates of *ρ* and *T*_*c*_ were similar to when the single full cross section was fitted to and not too dissimilar from when 2 cross-sections were fitted together. However the estimate of *λ* was much higher in 1-9s in comparison to the full single cross section and gamma was similar to when both cross-sections were fitted together, but lower than when all the data was evaluated.

When we fitted only 1-9 year olds the precision and accuracy of the estimated parameters was lower than when the all-age data was fitted to, therefore we evaluated whether sampling an additional age group outside of the current indicator group containing the same total number of samples could help improve the precision and accuracy of the parameter estimates. When including an additional age group outside of the current indicator group, for *λ* the precision and accuracy of the estimate when 20-30 year olds were also sampled was much improved, and the median estimate of 0.095 was very close to the true value of 0.10. The precision of the estimated value of *γ* was generally poorer than when only 1-9s were evaluated, however when a second group was also fitted to the accuracy of the estimate to the true value was much better than when only 1-9s were fitted to. Including age ranges above 30 years slightly reduced the precision of the estimate in comparison to when 10-20 or 20-30 year olds were included. Incorporating an additional age group up to 50 years of age helped improve the precision and accuracy of the estimated value of *ρ*, highlighting the value of sampling outside of the current indicator group for more precise parameter estimates. The most precise and accurate estimates of *ρ* were obtained when individuals 20-30 years were in the sample as well. *T*_*c*_ was also most accurately and precisely estimated when 20-30 year olds were included in the sample.

## Discussion

Inconsistencies in the observations from PCR and TF samples can make interpretation of trachoma surveillance data challenging [6]. The similarity observed between PCR and TF prevalence that breaks down as prevalence declines is currently not well understood or fully explained. In this article we have presented a possible explanation as to how these observations in surveillance data may be occurring. Through evaluating the proportion of individuals that would be present in each diagnostic state in the community with a dynamic model we have shown that as prevalence declines within a community the proportion of individuals PCR only or PCR and TF positive declines and a higher proportion of the PCR or TF positive population are only TF positive. These changes in the proportion of people that would test positive in each diagnostic state impact the diagnostic test results, making the proportion of TF and PCR positives less similar. The dynamics of transmission also mean that as prevalence declines the probability of detecting at least 1 positive individual by PCR with a fixed sample size declines much more rapidly than with TF (assuming a fixed sensitivity of the diagnostic over time). We note that an individual-based modelling approach would be needed to fully explain the observations seen in surveillance data. Importantly, individual-level diagnostic data from low prevalence settings would help us to understand whether the proportions of PCR and TF positives align with those predicted by the model. Individual level data is essential for testing the assumptions in this model and providing guidance on sampling strategies for PCR use in routine surveillance.

For sero-surveillance we have shown that much more accurate and precise parameter estimates can be inferred when 2 cross-sections are fitted to in comparison to 1. Particularly for the pre-intervention cross-section, we clearly saw how estimates of *λ* and *ρ* could be traded off with one another causing imprecise estimation [18]. When only fitting to data from 1-9 year olds the accuracy and precision of the parameter estimation was reduced in comparison to fitting to the all-age data. However, through the inclusion of one additional age-group we were able to improve the precision and accuracy of all parameter estimates when fitting 2 cross-sections simultaneously. In this situation it appeared that the inclusion of 20-30 year olds as well as 1-9 year olds had the most substantial impact on improving parameter estimation precision and accuracy. Therefore in terms of helping to quantify epidemiological parameters more accurately in the future we would suggest that at least 2 cross-sections be collected from the same community and that an age-group outside of the 1-9 year old group also be sampled. This will ensure that both *ρ* and the force of infection (determined by *λ*) are estimated more accurately and with less uncertainty.

For a number of NTDs the issue of systematic non-compliance/adherence to treatment has been reported and the potential issues it may present to elimination evaluated, ie. Treatment coverage within the community is not random. However, for the purposes of this study when modelling treatment we have assumed coverage is random. If individuals in the community systematically miss treatment then they may remain a reservoir source of infection, helping to ensure on-going transmission. However, for trachoma in particular little to no epidemiological data has been presented to suggest that systematic non-compliance is occurring during MDA rounds, and generally the coverage level is reported to be at least at the target level of 80%, as such, currently no data are available to indicate to what extent systematic non-compliance may be occurring. Despite our assumption of random coverage we do not expect a large impact at these coverage levels for the qualitative conclusions, unless it is quite extreme. However, if those being treated are the same as those being tested, and there were a group who were consistently not treated or measured, that would be more of a problem for the discussion posed here. Also, at these coverage levels, systematic non-compliance becomes a particular issue when non-adherence to treatment is correlated with infection risk, ie if those more at risk of infection continually miss treatment then they are more likely to remain a reservoir source of infection in the community. If this is the case in the communities we have evaluated, we would be more likely to see faster rates of re-emergence of infection but the qualitative observations and diagnostic outcomes reported in the study would be unlikely to change.

The are a number of limitations to the study. Firstly, for both diagnostic tests we assumed 100% specificity [17], if this assumption were relaxed we would expect an increase in the proportion of overall positives, leading to a possible over-estimate of the prevalence, and thus increasing the probability of detection with each diagnostic. However, despite modelling a slightly higher proportion of positives in the population in comparison to what may be true we would not expect the qualitative form of the relationships observed to be altered. Secondly it is possible that the sensitivity and specificity of the PCR and TF diagnostics may alter over time as prevalence declines [6], whereas we have only considered a single fixed value. Again, it is likely that this assumption would not alter the qualitative relationships observed here, but potentially the magnitude. As prevalence declines it becomes more challenging to detect both infection and disease, therefore we would expect the true probability of detection to potentially be even lower. Furthermore we would expect the noise around the low prevalence estimates to increase substantially [20]. Thirdly, in the sero-surveillance work, we chose to illustrate the importance of a second cross-section over only one, with an assumed reduction in transmission compared with 10 years previously, as we felt this was a case which would illustrate the point most effectively. However, as we approach an era of elimination for trachoma it is becoming increasingly unlikely that the opportunity will arise to conduct 2 surveys 10 years apart in time, therefore the question becomes how frequently should surveys be conducted in order to help accurately estimate the sero-reversion rate. This crucially depends on the rate of antibody decay, which is not yet known. However with exploratory simulation it may be possible to get a better idea on how frequently surveys should be conducted in order to estimate this. Lastly, in settings where urogenital infection is high such as the South Pacific [21], individuals may also test sero-positive to anti-trachoma antigens as a result of exposure to urogenital chlamydia. This can complicate the estimation of the sero-reversion and conversion rates for exposure due to trachoma, and would potentially need to be accounted for if sero-surveillance data was being collected in individuals past the age of sexual debut in populations with a high incidence of urogenital chlamydia infection.

PCR and sero-surveillance are important potential tools for trachoma surveillance, which may offer additional opportunities for understanding transmission dynamics as incidence declines. This paper highlights some of the links between these dynamics and potential survey design. However, there are, of course, many logistical constraints which would need to be considered before they were implemented widely in routine surveillance. From this study we highlight 2 key recommendations for future data collection for trachoma surveillance, in order to understand low-level transmission dynamics in greater detail as population prevalence declines. Firstly, individual-level diagnostic data from low prevalence settings would help us to understand whether the proportions of PCR and TF positives align with those predicted by the model. Secondly, we clearly highlight that for sero-surveillance more accurate and precise parameter estimates can be inferred when 2 cross-sections are fitted to in comparison to 1. We would therefore recommend at least 2 cross-sectional serological surveys being conducted several years apart in order to improve the estimation of epidemiological parameters from serological data.

## Supporting information

S1 File Code for the mathematical model in R. (ZIP)
